# Special Issue “Advanced Materials for Water Remediation”

**DOI:** 10.3390/ma15155096

**Published:** 2022-07-22

**Authors:** Gabriela Buema, Oana-Georgiana Dragos-Pinzaru, Horia Chiriac, Nicoleta Lupu, Daniel Gherca

**Affiliations:** National Institute of R&D for Technical Physics, 700050 Iasi, Romania; gbuema@phys-iasi.ro (G.B.); odragos@phys-iasi.ro (O.-G.D.-P.); hchiriac@phys-iasi.ro (H.C.); nicole@phys-iasi.ro (N.L.)

“Advanced Materials for Water Remediation” is a Special Issue of *Materials*, which will take into consideration all the papers discussing the synthesis, characterization and application of advanced materials for water remediation. The development of cost-effective and stable advanced materials, strategies and technology for providing pure water is a critical need for environmental protection. It is well recognized that nanoscience and nanotechnology have made a revolutionary impact on the scientific community with many new interesting research fields. Nanotechnology addresses the continuous development of solutions to the existing environmental problems, and preventive measures for future problems. However, nanoscience developments facilitate a number of emerging technologies to be addressed to solve the multiple problems of water in order to ensure the environmental stability and finally assisting the attainment of water quality standards and health advisories. New applications and new products emerge in many different fields, i.e., energy, environment, medicine, and these developments will most probably change our society.

Water is a natural and essential resource for sustaining all forms of life on Earth. Water pollution (defined as alteration in the physical, chemical and biological properties) is an increasingly dangerous problem, representing currently one of the most important environmental risk to human health, affecting people across the world. The precipitation, ion exchange, adsorption, membrane separation, reverse osmosis, and electrocoagulation are some of the methods that were suggested in order to remove the pollutants. The most effective method used to date for removing different pollutants is the adsorption method [1]. It must be emphasized that the successful remediation of water by adsorption technique strongly depends on the material used [2].

The metals with a density higher than 5 g/cm^3^ are considered toxic heavy metals. Among the most harmful heavy metals are cadmium and chromium. Due to its carcinogenic properties, cadmium may be hazardous to human health [3]. Layered double hydroxide (LDH) in its raw, calcined or modified forms with Fe_3_O_4_ provides a solution for wastewater treatment contaminated with cadmium ion [4]. On the other hand, a study conducted by Buema et al. proposes a new type of advanced composite material with improved adsorption characteristics based on fly ash/NaOH along with the advantage of a simple and low-cost method of synthesis [5]. Recently, Payel and co-workers reported a study regarding the chromium adsorption by recycling biochar derived from tannery liming sludge. They demonstrated that, in static and dynamic conditions, chromium adsorption reached 152.1 mg/g and 533.4 mg/g, respectively [6].

Copper and Cobalt ions are also recognized as toxic metals that can polluted the waters. Copper ion is used for different applications, in various fields, such as: fertilizer, paints, plating baths, and paper goods [7]. An industrial waste (fly ash) treated with NaOH or Fe_3_O_4_ can be involved as an adsorbent for copper ion removal [8,9,10]. Cobalt ion, with applications in nuclear power plants, metallurgy, mining, pigments, paints, electronic, and electroplating industries, can lead to diseases, such as: asthma-like allergy, damage to the heart, causing heart failure, and damage to the thyroid and liver. Various materials were used as adsorbents in order to remove this contaminant. These studies present the effect of different working parameters that affect the cobalt adsorption process, such as: pH, adsorbent dose, initial cobalt concentration, contact time, and temperature [11,12,13,14].

Other contaminants dangerous for the environment are uranium and europium. The safe and acceptable level for the carcinogenic risk from uranium, according to the USEPA, is zero tolerance, taking into account that this pollutant is a carcinogen of category A [15]. One of the most significant radionuclides found in radioactive waste that is emitted accidently or often is europium [16]. Their removal is mentioned in different studies using winery wastes, raw/modified pomegranate peel, and carbon-modified zirconia/spinel ferrite nanostructures as adsorbents [15,16,17].

An important source of contaminants of the aquatic environments is represented by the dyes, the largest amount coming from the textile industry. The production and use of these dye compounds may not be safe, as evidenced by the hazardous characteristics of some of them, including their potential for cancer, allergic reactions, and dermal impacts, according to available literature data [18]. The main investigated dyes presented in the specialized literature are: Methylene blue, Rhodamine B, Congo Red, Methyl orange, Safranin, etc.

Phenolic compounds are the most common organic contaminants in industrial effluent: including p-nitrophenol (4-NP), phenol, and 4-chlorophenol (4-CP) [19].

4-NP comes from the pharmaceutical and chemical industries. The techniques proposed for its elimination consist of advanced oxidative processes, biodegradation, chemical reduction, and solvent extraction. The chemical reduction process is recommended taking into account that this method presents the advantage that the reaction’s by-product is itself a valuable product that serves as a lubricant against corrosion, a medication intermediary, or a photographic developer. The chemical reduction process occurs in presence of catalysts, so the efficient catalysts able to increase the efficiency of the reduction reaction of aromatic nitro-compounds, should be developed. Due to its simplicity, researchers frequently employ the 4-NP reduction to 4-aminophenol (4-AP) by sodium borohydride NaBH_4_ reaction [20,21]. Pt-group metals in forms of rods, wires prisms, or branched nanostructures, are typically utilized as catalysts for the hydrogenation of nitro compounds. Additionally, the literature presents the possibility to involve the adsorption process for the removal of 4-NP using different materials: steel slag (with an adsorption capacity of 109.66 mg/g) [22], and cyclodextrin polymer-entrapped nano zero-valent iron [23]. A composite prepared from clay and Cocos nucifera shell was proposed by Adebayo and Areo for the removal of phenol and 4-NP. Their study demonstrates that the material proposed shows higher adsorption capacities values: 1665 mg/g (phenol) and 476.9 mg/g (4-nitrophenol), respectively [24]. Lunagariya and co-workers demonstrated that with the aim to increase the effectiveness of phenol removal, Taguchi method is a promising, effective, and economical strategy for the industry [25]. On the other hand, Dehmani et al. present a review of phenol adsorption on transition metal oxides. The adsorption capacity of phenol using different adsorbents reported in the literature is also presented [26].

Sarno and Iuliano present an interesting study regarding a nano-biocatalyst for 4-CP removal from wastewater. The degradation of 4-CP in the presence of the proposed material was 98% after 180 minutes of contact time [27]. Lei et al. studied the simultaneous removal of 4-CP and chromium ions. Their findings highlighted that the composite material comprising polypyrrole-supported Pd/Fe nanoparticles completely removes 4-CP and chromium [28].

Another class of emerging contaminants are per- and polyfluoroalkyl substances (PFAS). People can present various diseases if they are exposed to PFAS. The research team formed by Das and Ronen shows in their excellent review a series of methods used in order to remove these substances from wastewaters [29]. Belkouteb and co-workers demonstrate that granular activated carbon (GAC) filters offer a technique for removing PFASs [30]. A material based on cellulose fibers functionalized with quaternized wood pulp has the capacity to remove one of the most prevalent PFASs substances: 763 mg/g of perfluorooctanesulfonic acid (PFOS) and 605 mg/g of perfluorooctanoic acid (PFOA) [31].

The data obtained through the adsorption process are modelled using isotherms (i.e., Langmuir, Freundlich, Temkin, Sips, Redlich Peterson, and Dubinin–Radushkevich) and kinetics models (Pseudo first order, Pseudo second order, Intraparticle diffusion) in their linear or nonlinear forms. For the purpose of designing the adsorption systems, adsorption isotherm models provide information on the adsorption process. The thermodynamic study including Gibbs free energy change (ΔG°), enthalpy change (ΔH°), and entropy change (ΔS°) is presented in order to confirm the nature of the adsorption process.

Moreover, it is essential to conduct aquatic toxicity tests in order to evaluate the water quality for human consumption and forecast the effects of pollutants on ecosystems [32]. For example, a long-used method is daphnia magna test, which is applicable to: chemical substances that are soluble under the conditions of the test; industrial/sewage effluents; untreated and treated wastewaters; aqueous extracts and leachates; and eluates of fresh water sediment. For example, Daphnia magna as a measure of the toxicity and effectiveness of treating textile wastewaters was investigated by Villegas-Navarro and co-workers [33]. Additionally, Sakai applied this technique for examination of river water quality [34]. Through this editorial, we also encourage laboratory studies on real wastewaters so that we can move forward by approaching the discovery of as many influences as possible.

It is also well known that waste management is an important tool for achieving a circular economy (CE) [35]. The CE, a model of production and consumption, represents a sustainable alternative whose main concepts are: (i) how to manage resources; (ii) how to make and use products; and (iii) what to do with the materials afterwards. 

Acevedo-García and co-workers proposed a study based on synthesis and applicability of adsorbents using the principles of CE [36]. The biochar derived from the lime fiber waste was employed for the adsorption of a mixture with sulfamethoxazole (SMX) and methyl paraben (MP). Mladenovic and co-workers studied a CE approach for rice husk modification, with accent on equilibrium, kinetic, thermodynamic studies, and mechanism of Congo red dye removal [37]. Their findings demonstrated the CE concept’s applicability in the development of an effective adsorbent without wasting more chemicals or energy. An examination of the utilization of biowaste as a biosorbent for the removal of heavy metals by CE point of view is presented by Madea and Skuza [38]. The studies regarding the concept of CE in metal recovery were also completed by Ahmed and co-workers. The authors mentioned that the standard “take-make-consume-and-dispose” model has limitations, which has led to the development of the CE idea. They investigated the removal of toxic pollutants from wastewater and water reuse within a circular economy [39].

The main goal of the study proposed by Barros and colleagues was the development of an environmentally friendly technology for the treatment of wastewater contaminated with different rare earth elements, such as lanthanum, cerium, yttrium, terbium, praseodymium, and europium, from a CE perspective [40].

In conclusion, this concept must be implemented as much as possible, taking into account that its idea is focused on recycling and reusing materials.

A recyclability study of the materials, which is an important point for practical applications, is recommended using different reagents, i.e., NaCl, HNO_3_, HCl, and Na_2_CO_3_. Furthermore, the adsorbent loaded with a contaminant is analyzed using different techniques (SEM, EDX, FTIR, XPS, and TGA) in order to confirm the interaction of the pollutant with the surface of the adsorbent. Based on the information obtained, an adsorption mechanism is provided.

In order to prove the safe disposal of the loaded adsorbents into the environment, ‘’Toxicity Characteristic Leaching Procedure’’ (TCLP protocol) can be performed [5].

In general, as a consequence of the diversity in requirements for different applications, it becomes very difficult to devise generalized guidelines on what makes “the best” material, and that is why this Special Issue aims to make as significant contributions as possible to this field, which has manifested itself in a diverse spectrum of research directions and emerging applications. To this end and to meet the challenges of the 21st century that will hopefully open new horizons for the valorization of multifunctional materials at the nanoscale level, the scope of this Special Issue is to design and develop, using environmentally friendly processes, nano-organized heterostructures for innovative high-added value applications (water remediation, heavy metal and dyes adsorption).

In conclusion, this Special Issue aims to add value to the existing literature by proposing advanced materials with the applicability for treatment of waters contaminated with different pollutants, including heavy metals, dyes, phenolic compounds, etc. It is our hope that the Special Issue “Advanced Materials for Water Remediation” of *Materials* will deliver scientific research outcomes that (i) will be devoted to the knowledge-based economy of Europe; (ii) will represent an important contribution for the new strategic context of the European Green Deal future initiative to ensure a toxic-free environment before 2050; (iii) will address, on a global scale, a highly relevant environmental issue included in “Clean Water and Sanitation” of the UN Sustainable Development Goals and (iv) will raise the competitiveness and excellence of the Global/European Research Area.

## References

[1] Han X., Li R., Miao P., Gao J., Hu G., Zhao Y., Chen T. (2022). Design, Synthesis and Adsorption Evaluation of Bio-Based Lignin/Chitosan Beads for Congo Red Removal. Materials.

[2] Zhang W., Liang Y., Wang J., Zhang Y., Gao Z., Yang Y., Kai Yang K. (2019). Ultrasound-assisted adsorption of Congo red from aqueous solution using MgAlCO_3_ layered double hydroxide. Appl. Clay Sci..

[3] Lan Z., Lin Y., Yang C. (2022). Lanthanum-iron incorporated chitosan beads for adsorption of phosphate and cadmium from aqueous solutions. Chem. Eng. J..

[4] Gherca D., Borhan A.I., Mihai M.M., Herea D.D., Stoian G., Roman T., Chiriac H., Lupu N., Buema G. (2022). Magnetite-inducedtopological transformation of 3D Hierarchical MgAl layered double hydroxides to highly dispersed 2D magnetic hetero-nanosheets for effective removal of cadmium ions from aqueous solutions. Mater. Chem. Phys..

[5] Buema G., Lupu N., Chiriac H., Ciobanu G., Bucur R.D., Bucur D., Favier L., Harja M. (2021). Performance assessment of five adsorbents based on fly ash for removal of cadmium ions. J. Mol. Liq..

[6] Payel S., Hashem M.A., Hasan M.A. (2021). Recycling biochar derived from tannery liming sludge for chromium adsorption in static and dynamic conditions. Environ. Technol. Innov..

[7] Azam M., Wabaidur S.M., Khan M.R., Al-Resayes S.I., Islam M.S. (2022). Heavy Metal Ions Removal from Aqueous Solutions by Treated Ajwa Date Pits: Kinetic, Isotherm, and Thermodynamic Approach. Polymers.

[8] Buema G., Harja M., Lupu N., Chiriac H., Forminte L., Ciobanu G., Bucur D., Bucur R.D. (2021). Adsorption performance of modified fly ash for copper ions removal from aqueous solution. Water.

[9] Buema G., Trifas L.-M., Harja M. (2021). Removal of Toxic Copper Ion from Aqueous Media by Adsorption on Fly Ash-Derived Zeolites: Kinetic and Equilibrium Studies. Polymers.

[10] Harja M., Buema G., Lupu N., Chiriac H., Herea D.D., Ciobanu G. (2021). Fly Ash Coated with Magnetic Materials: Improved Adsorbent for Cu (II) Removal from Wastewater. Materials.

[11] Abdelfatah A., Abdel-Gawad O.F., Elzanaty A.M., Rabie A.M., Mohamed F. (2021). Fabrication and optimization of poly(ortho-aminophenol) doped glycerol for efficient removal of cobalt ion from wastewater. J. Mol. Liq..

[12] Acosta-Rodríguez I., Rodríguez-Pérez A., Pacheco-Castillo N.C., Enríquez-Domínguez E., Cárdenas-González J.F., Martínez-Juárez V.-M. (2021). Removal of Cobalt (II) from Waters Contaminated by the Biomass of Eichhornia crassipes. Water.

[13] Wang R., Deng L., Fan X., Li K., Lu H., Li W. (2021). Removal of heavy metal ion cobalt (II) from wastewater via adsorption method using microcrystalline cellulose–magnesium hydroxide. Int. J. Biol. Macromol..

[14] Savastru E., Bulgariu D., Zamfir C.-I., Bulgariu L. (2022). Application of Saccharomyces cerevisiae in the Biosorption of Co(II), Zn(II) and Cu(II) Ions from Aqueous Media. Water.

[15] Noli F., Kapashi E., Pashalidis I., Margellou A., Karfaridis D. (2022). The effect of chemical and thermal modifications on the biosorption of uranium in aqueous solutions using winery wastes. J. Mol. Liq..

[16] Abdel Maksoud M.I.A., Sami N.M., Hassan H.S., Bekhit M., Ashour A.H. (2022). Novel adsorbent based on carbon-modified zirconia/spinel ferrite nanostructures: Evaluation for the removal of cobalt and europium radionuclides from aqueous solutions. J. Colloid Interface Sci..

[17] Noli F., Busari Nasiru M.S.A., Tsamos P., Pavlidou E. (2022). Eu(III) removal from aqueous solutions using raw and modified pomegranate peel as biosorbents. Int. J. Environ. Sci. Technol..

[18] Tkaczyk A., Mitrowska K., Posyniak A. (2020). Synthetic organic dyes as contaminants of the aquatic environment and their implications for ecosystems: A review. Sci. Total Environ..

[19] Zhao C., Xue L., Shi H., Chen W., Zhong Y., Zhang Y., Zhou Y., Huang H. (2022). Simultaneous degradation of p-nitrophenol and reduction of Cr(VI) in one step using microwave atmospheric pressure plasma. Water Res..

[20] Hashimi A.S., Nohan M.A.N.M., Chin S.X., Zakaria S., Chia C.H. (2019). Rapid Catalytic Reduction of 4-Nitrophenol and Clock Reaction of Methylene Blue using Copper Nanowires. Nanomaterials.

[21] Grzeschik R., Schäfer D., Holtum T., Küpper S., Hoffmann A., Schlücker S. (2020). On the Overlooked Critical Role of the pH Value on the Kinetics of the 4-Nitrophenol NaBH_4_-Reduction Catalyzed by Noble-Metal Nanoparticles (Pt, Pd, and Au). J. Phys. Chem. C.

[22] Zhao Y., Wang L., Zhu L., Gao F., Xu X., Yang J. (2022). Removal of p-Nitrophenol from simulated sewage using steel slag: Capability and mechanism. Environ. Res..

[23] Yang X., Yu S., Wang M., Liu Q., Jing X., Xiyun Cai X. (2022). One-pot preparations of cyclodextrin polymer-entrapped nano zero-valent iron for the removal of p-nitrophenol in water. Chem. Eng. J..

[24] Adebayo M.A., Areo F.I. (2021). Removal of phenol and 4-nitrophenol from wastewater using a composite prepared from clay and Cocos nucifera shell: Kinetic, equilibrium and thermodynamic studies. Resources. Environ. Sustain..

[25] Lunagariya J., Chabhadiya K., Pathak P., Mashru D. (2022). Application of Taguchi method in activated carbon adsorption process of phenol removal from ceramic gasifier wastewater. Environ. Chall..

[26] Dehmani Y., Dridi D., Lamhasni T., Abouarnadasse S., Chtourou R., Lima E.C. (2022). Review of phenol adsorption on transition metal oxides and other adsorbents. J. Water Process. Eng..

[27] Sarno M., Iuliano M. (2020). New nano-biocatalyst for 4-chlorophenols removal from wastewater. Mater. Today Proc..

[28] Lei C., Zhou Z., Chen W., Xie J., Huang B. (2022). Polypyrrole supported Pd/Fe bimetallic nanoparticles with enhanced catalytic activity for simultaneous removal of 4-chlorophenol and Cr(VI). Sci. Total Environ..

[29] Das S., Ronen A. (2022). A Review on Removal and Destruction of Per-and Polyfluoroalkyl Substances (PFAS) by Novel Membranes. Membranes.

[30] Belkouteb N., Franke V., McCleaf P., Köhler S., Ahrens L. (2020). Removal of per- and polyfluoroalkyl substances (PFASs) in a full-scale drinking water treatment plant: Long-term performance of granular activated carbon (GAC) and influence of flow-rate. Water Res..

[31] Harris J.T., de la Garza G.D., Devlin A.M., McNeil A.J. (2022). Rapid Removal of Poly- and Perfluoroalkyl Substances with Quaternized Wood Pulp. ACS EST Water.

[32] Huang Y., Campana O., Wlodkowic D. (2017). A Millifluidic System for Analysis of Daphnia magna Locomotory Responses to Water-born Toxicants. Sci. Rep..

[33] Villegas-Navarro A., Romero González M.C., Rosas López E., Domínguez Aguilar R., Sachetin Marçal W. (1999). Evaluation of Daphnia magna as an indicator of toxicity and treatment efficacy of textile wastewaters. Environ. Int..

[34] Sakai M. (2001). Chronic toxicity tests with Daphnia magna for examination of river water quality. J. Environ. Sci. Health B.

[35] Snellinx S., Van Meensel J., Farahbakhsh S., Bourgeois L., Mertens A., Lauwers L., Jeroen Buysse J. (2021). Waste treatment company decision-making in a complex system of markets influenced by the circular economy. J. Clean. Prod..

[36] Acevedo-García V., Rosales E., Puga A., Pazos M., Sanromán M.A. (2020). Synthesis and use of efficient adsorbents under the principles of circular economy: Waste valorisation and electroadvanced oxidation process regeneration. Sep. Purif. Technol..

[37] Mladenovic N., Petkovska J., Dimova V., Dimitrovski D., Jordanov I. (2022). Circular economy approach for rice husk modification: Equilibrium, kinetic, thermodynamic aspects and mechanism of Congo red adsorption. Cellulose.

[38] Madeła M., Skuza M. (2021). Towards a Circular Economy: Analysis of the Use of Biowaste as Biosorbent for the Removal of Heavy Metals. Energies.

[39] Ahmed M., Mavukkandy M.O., Giwa A., Elektorowicz M., Katsou E., Khelifi O., Naddeo V., Hasan S.W. (2022). Recent developments in hazardous pollutants removal from wastewater and water reuse within a circular economy. NPJ Clean Water.

[40] Barros Ó., Costa L., Costa F., Lago A., Rocha V., Vipotnik Z., Silva B., Tavares T. (2019). Recovery of Rare Earth Elements from Wastewater Towards a Circular Economy. Molecules.

